# Central defect type partial ACL injury model on goat knees: the effect of infrapatellar fat pad excision

**DOI:** 10.1186/s13018-015-0281-x

**Published:** 2015-09-04

**Authors:** Bekir Karakilic, Emin Taskiran, Basak Doganavsargil, Bora Uzun, Salih Celik, Elcil Kaya Bicer

**Affiliations:** Orthopaedics and Traumatology Clinic, Bayburt State Hospital, Bayburt, Turkey; Orthopaedics and Traumatology Department, Ege University Hospital, Izmir, Turkey; Pathology Department, Ege University Hospital, Izmir, Turkey; Biomechanics Department, Dokuz Eylul University, Izmir, Turkey

**Keywords:** Partial ACL injury model, Primary healing, Infrapatellar fat pad

## Abstract

**Background:**

The mid-substance central defect injury has been used to investigate the primary healing capacity of the anterior cruciate ligament (ACL) in a goat model. The sagittal plane stability on this model has not been confirmed, and possible effects of fat pad excision on healing have not been evaluated. We hypothesize that excising the fat pad tissue results in poorer ligament healing as assessed histologically and decreased tensile strength of the healing ligament. We further hypothesize that the creation of a central defect does not affect sagittal plane knee stability.

**Methods:**

A mid-substance central defect was created with a 4-mm arthroscopic punch in the ACLs of right knees of all the subjects through a medial mini-arthrotomy. Goats were assigned to groups based on whether the fat pad was preserved (group 1, *n* = 5) or excised completely (group 2, *n* = 5). The left knees served as controls in each goat. Histopathology of the defect area along with measurement of type I collagen in one goat from each group were performed at 10th week postoperatively. The remaining knees were evaluated biomechanically at the 12th week, by measuring anterior tibial translation (ATT) of the knee joints at 90° of flexion and testing tensile properties (*ultimate tensile load* (*UTL*), *ultimate elongation* (*UE*)*, stiffness* (*S*), *failure mode* (*FM*)) of the femur-ACL-tibia complex.

**Results and discussion:**

Histopathology analysis revealed that the central defect area was fully filled macroscopically and microscopically. However, myxoid degeneration and fibrosis were observed in group 2 and increased collagen type I content was noted in group 2. There were no significant differences within and between groups in terms of ATT values (*p* = 0.715 and *p* = 0.149, respectively). There were no significance between or within groups in terms of ultimate tensile load and ultimate elongation; however, group 2 demonstrated greater stiffness than group 1 that was correlated with the fibrotic changes detected microscopically (*p* = 0.043).

**Conclusions:**

The central defect type injury model was confirmed to be biomechanically stable in a goat model. Resection of the fat pad was noted to negatively affect defect healing and increase ligament stiffness in the central defect injury model.

## Introduction

The anterior cruciate ligament (ACL) has an important role in knee joint stability. Injuries in active individuals can jeopardize future athletic function and lead to degenerative arthritis. These concerns have led to many the studies on surgical reconstruction of the ACL in recent years, but there is still no gold standard treatment proven to reliably restore function and prevent arthritis. The primary healing capacity of ACL tissue is limited compared to other ligaments such as the medial collateral ligament (MCL) due to differences in vascularity, biological environment, and mechanical processes [1–5]. These influences on these factors and potential interventions can be effectively studied with animal injury models [6–16]. The central defect type partial ACL injury model described by Murray et al is thought to be a mechanically stable model, which enables concentration on healing and repair processes [6, 10, 11]. The goat knee was selected as suitable animal model due to its anatomical and mechanical similarities to the human knee joint [17–19] and that has been utilized in multiple prior studies [20–33].

The infrapatellar fat pad has been shown to have an important role in ACL circulation [34–36]. However, in previous studies using the central defect model, the injury was created without regard to protection of the fat pad tissue. Further, although the model has been assumed to be mechanically stably in the sagittal plane, this stability has not been confirmed to mechanical testing.

In this study we aimed to investigate the primary healing capacity of the ACL using central defect type partial injury model and evaluate the effect of fat pad excision on ligament histology and strength. We also aim to confirm the sagittal plan stability of the central defect model. We hypothesize that excising fat pad tissue results in poorer ligament healing as assessed histologically and decreased tensile strength of the healing ligament. We further hypothesize that the creation of a central defect does not affect sagittal plane knee stability.

## Methods

### Study groups

Ten adolescent female Anatolian Black Goats aged between 7 and 11 months were included in the study. Approval and permission from Ege University Animal Care and Use Ethic Committee was obtained for the study. All animals were anesthetized with a ketamine-xylazine combination by a specialist veterinary physician. Five animals were assigned to each of two groups. In group 1, a central defect was created in the ACL of the right knee joints of the animal without any harm to the fat pad tissue. In group 2, the same ACL injury model was performed after complete excision of the infrapatellar fat pad tissue. Left knee joints were left as controls without any intervention.

### Surgical technique

**Fig. 1:**
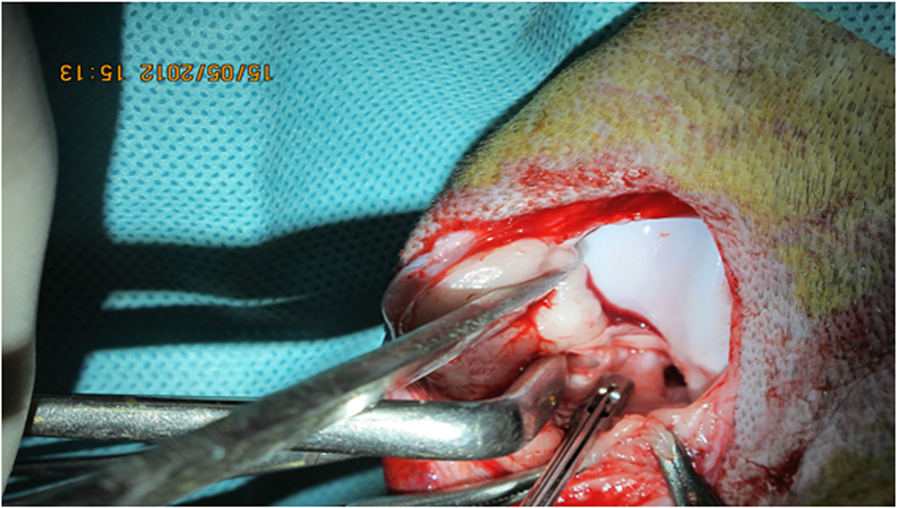
Creating central defect in ACL

**Fig. 2:**
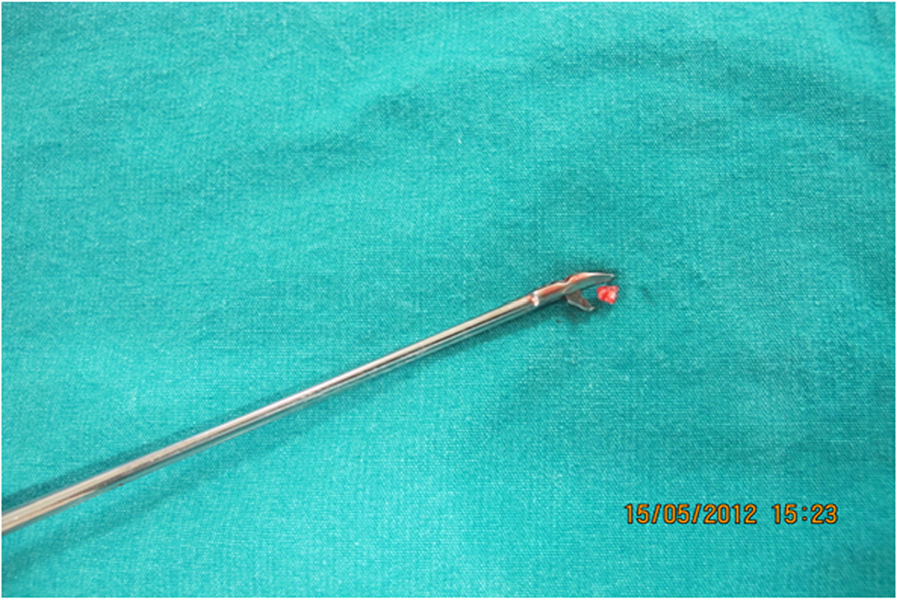
Excised full thicknees tissue from ACL for central defect

**Fig. 3:**
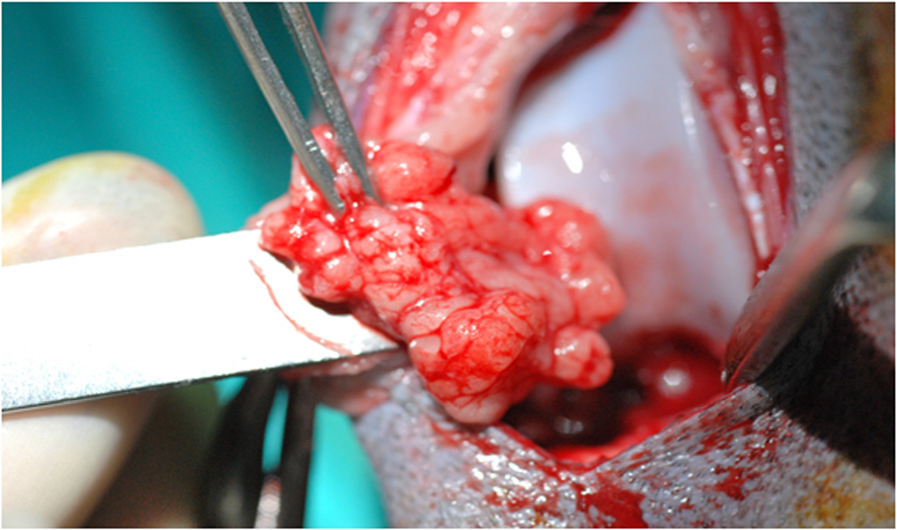
Fat pad excision

All knee joints of the animals were prepared under sterile conditions. To decrease intraoperative bleeding and gain clear exposure, subcutaneous jetocaine was injected before the skin incision. A medial parapatellar skin incision approximately 5 cm long was created, and a medial mini-arthrotomy was performed to access the joint in both groups. In group 1, the infrapatellar fat pad and synovial folds were protected and gently retracted to visualize the ACL. A central defect was then created by excising full thickness ligament tissue with a 4-mm arthroscopic punch (Figs. 1 and 2). In group 2, complete excision of the infrapatellar fat pad tissue was performed before creating the same central defect type injury in ACL (Fig. 3). After irrigating the knee joint, the arthrotomy and skin incision were closed in the same manner in both groups.

### Postoperative care

All animals were restricted to cage activity for 6 weeks after surgery. Analgesia (Metamizole, 20 mg/kg twice daily) and antibiotic prophylaxis (penicillin, 20.000 IU/Kg twice daily) were given during the early postoperative period (48 h). The surgical wounds were closed completely in 10 days. After 6 weeks, the animals were allowed daily activities out of the cages.

Two animals (one from each group) that were chosen at random for histopathology were sacrificed with overdose of pentobarbital at week 10, and the remaining animals were sacrificed at week 12 for mechanical testing. The hind limbs were amputated through the mid-shaft of the femur and tibia and kept at −20 °C until mechanical testing.

### Histopathological and immunohistochemical evaluation

Histopathology was performed on the operated and control knees of one randomly selected animal from each group sacrificed during week 10. Macroscopic evaluation was performed to detect the fill of the defect as well as to evaluate for any cartilage lesions. Histological analysis was performed to compare the samples for cellular and vascular responses. Immunohistochemistry was performed to compare the amount of type I collagen at the injury sites.

The samples from the sacrificed animals were stored at 4 °C. The knee joints were carefully dissected. The ACL injury area in the experimental knees and the corresponding area in the control knees were resected and fixed in 10 % formaldehyde solutions for 24 h. Four to five micrometric paraffin blocks were prepared and stained with hematoxylin eosin. Immunohistochemistry assessment for type I collagen was performed in frozen sections taken by cryomicrotome. After an antigen-producing stage with baking (EDTA pH, 8 5 min/850 W microwave), the samples were stained with monoclonal antibody for collagen-1 (*collagen 1 antibody*; *5D8-G9/Coll-NOVUS*) and kept at 4 °C for 24 h. Final labeling was performed with the streptoavidin-biotin method using dab chromogen. Kidney tissue was used for positive comparison.

### Mechanical testing

**Fig. 4:**
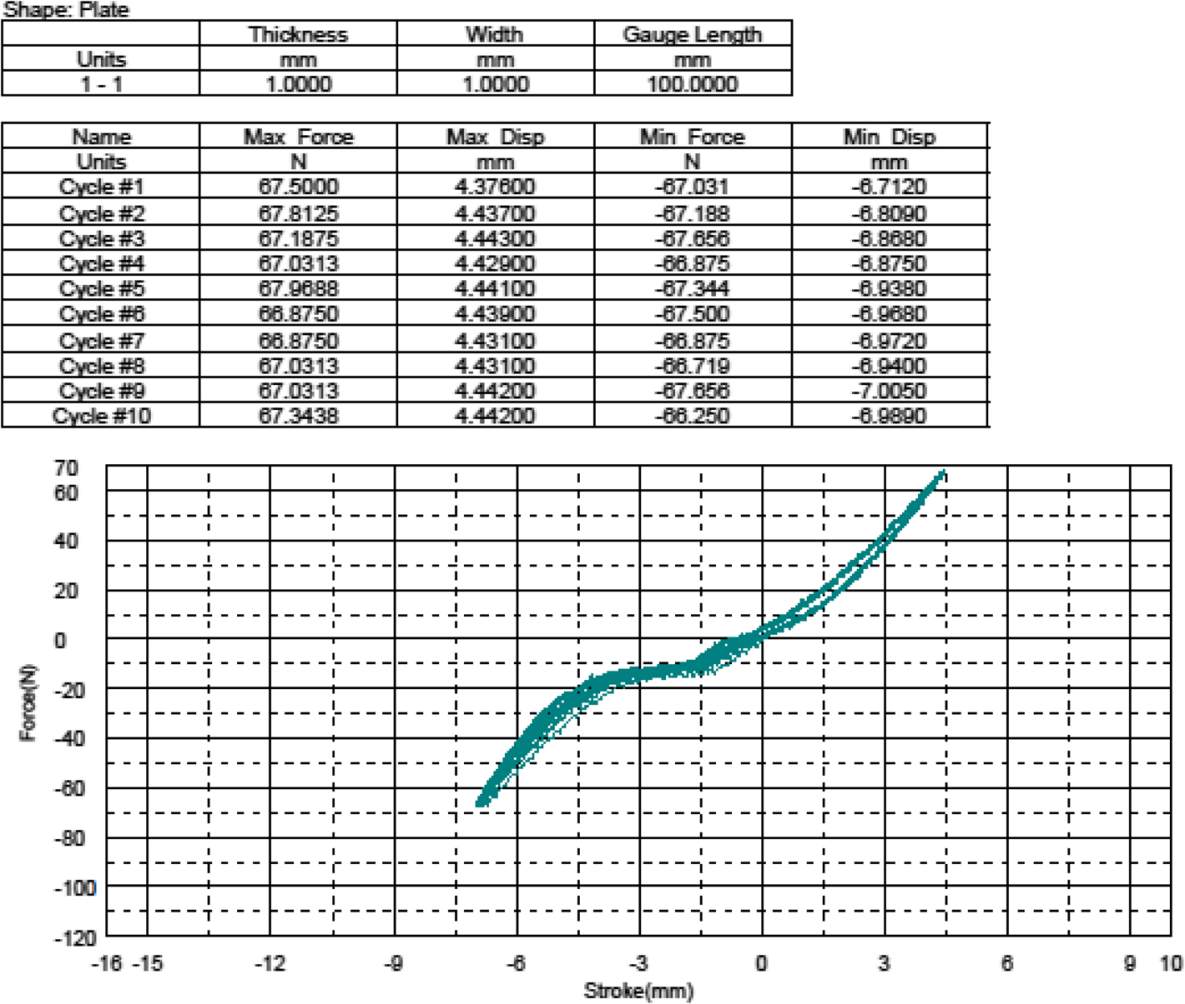
Anterior tibial translation test data of a sample

**Fig. 5:**
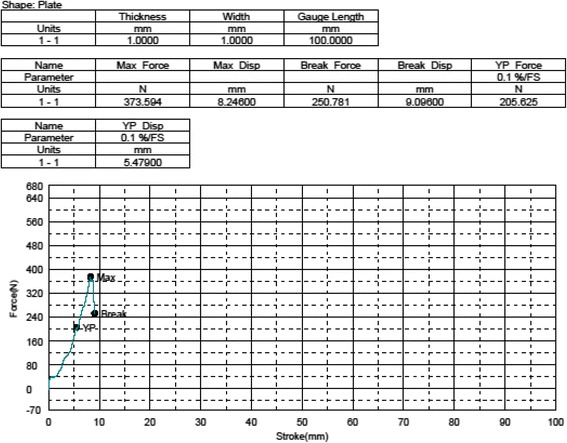
Tensile load to failure data of a sample

We measured the structural properties of the ligament as well as sagittal joint laxity (anterior tibial translation) to which the ACL is the primary contributor. A *Shimadzu AG 10 K* mechanical testing machine was used. In order to attach the joint samples to the machine, a custom device was manufactured as in the previous biomechanical studies [19, 37]. The knee joints of four animals in each group were tested. The previously amputated and frozen limb samples were allowed to thaw at room temperature 12 h before testing. After complete thawing, the soft tissues 5 cm proximal and distal to the joint line were dissected carefully preserving surrounding the soft tissues and joint capsule. The bones were embedded in cylindrical molds with bone cement. To measure anterior tibial translation, the cylindrical molds holding the knee samples were put into the cylindrical parts of the manufactured device and the whole system was located and fixed to the test machine orienting the tibia vertical and the knee flexed to 90°. The joint was cyclically loaded up to 67 Newtons (N) anteriorly and posteriorly at a rate of 20 mm/min (min) in order to simulate the in situ load born by the ACL in goats according to prior work [38]. The mean anterior displacement of 10 cycles of loading was recorded as the *mean anterior tibial translation value* of the specimens (Fig. 4). After finishing the anterior tibial translation (ATT) test, the specimens were taken out of the system. The remaining soft tissues including the capsule, menisci, collateral ligaments, and posterior cruciate ligament (PCL) were excised to obtain the femur-ACL-tibia complex. The femur-ACL-tibia complex were located and fixed to the test machine again for tensile tests. After pre-tensioning (5 N for 10 min) and pre-conditioning (10 cycles of load up to 20 N) stages, each specimen was tested to failure at 20 mm/min. Ultimate tensile load (*Newton*), *ultimate elongation* (*mm*), *stiffness* (*N/meter*), and *failure locations* were recorded (Fig. 5).

### Statistical analysis

Statistical analysis was performed utilizing SPSSv18 package program. *Mann–Whitney U* and *Wilcoxon’s signed rank test* were used for inter- and intragroup analysis, respectively. The level of statistical significance was set at *p* < 0.05.

## Results

### Histopathology and immunohistochemistry findings

**Fig. 6:**
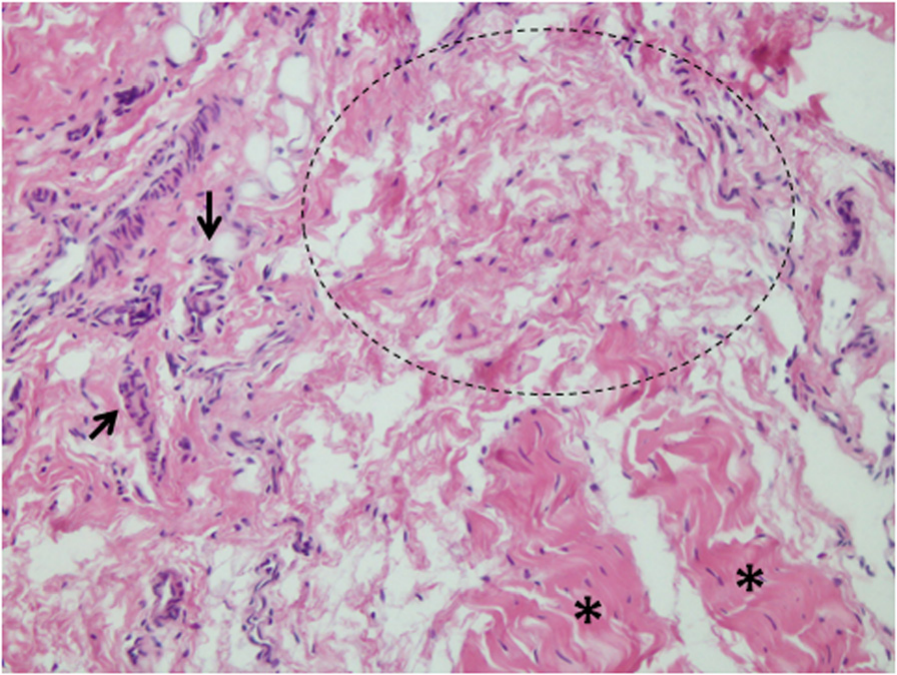
Microscopic section showing the filled defect area surrounded by vascular proliferation (*arrows*) and normal ligament tissue (*stars*)

**Fig. 7:**
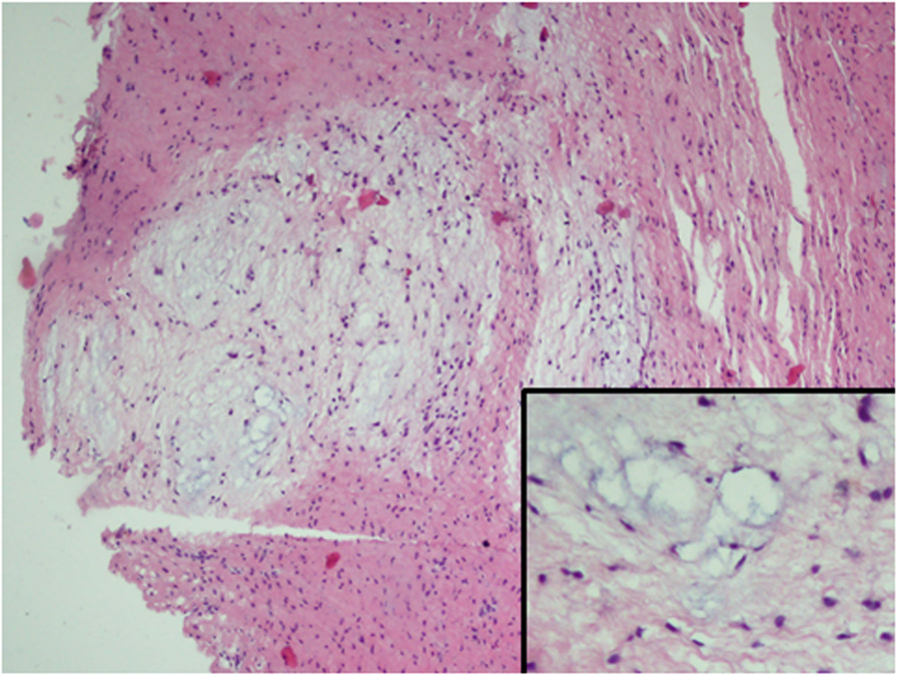
Microscopic section showing the defect area of fat-pad excised sample: having edema and myxoid degeneration fields

Macroscopic evaluations of all knees revealed full filling of injury site (Fig. 6). No cartilage lesion was detected among these samples. Microscopic evaluations showed more fibroblastic cells in the injured ACLs compared to their controls. No inflammatory cells were observed (Table 1). More vascular structures were detected surrounding the defect area in group 2 (fad pad excised). Histological assessment of this group also showed edematous changes and myxoid degeneration in the ligament injury site (Fig. 7). In contrast, normal histological healing of the ACL injury site was observed in group 1 (fat pad protected). More type 1 collagen stained fibers were detected in the injury site of the fat pad excised sample (Table 2).

**Table 1:** Vascular structures per micro observation site

Sample	Vascular count
A3—Experiment knee	25
A3—Control	11
B1—Experiment knee	46
B1—Control	25

**Table 2:** Collagen type 1 stained fiber count

Sample	Fiber count
A3—Experiment knee	69
A3—Control	68
B1—Experiment knee	88
B1—Control	42

### Biomechanical findings

#### ATT findings

No significant differences (group 1, p = 0.715; group 2, p = 0.715) (Tables 3 and 4) were found in sagittal joint laxity between the injured and control knee in either group. Fat pad excision had no effect on sagittal joint laxity (p = 0.149)

**Table 3:** Group A mean ATT values (mm)

Subject	Experiment knee	Control knee
A-1	6.4117	0.8788
A-2	4.1572	2.8145
A-4	3.1554	5.907
A-5	3.3138	4.4311

*A-3* undergone pathologic assessment

**Table 4:** Group B mean ATT values (mm)

Subject	Experiment knee	Control knee
B-2	2.613	4.4991
B-3	3.4019	2.1616
B-4	3.1074	1.9328
B-5	3.2669	2.9025

*B-1* undergone pathologic assessment

#### Tensile loading findings

Injured knee samples did not show any significant difference from their controls in tensile loadings in terms of ultimate tensile load (Tables 5 and 6). Stiffness values of the injured knees with fat pad excision were significantly higher than the fat pad preserved samples (*p* = 0.043). Tibial avulsion was the most common mode of failure among the samples in the tensile load analyses (9 of 16 knees)

**Table 5:** Group A tensile test values

Subject	Ultimate tensile load (N)		Ultimate elongation (mm)		Stiffness (N/meter)		Failure mode	
	Experiment knee	Control knee	Experiment knee	Control knee	Experiment knee	Control knee	Experiment knee	Control knee
A-1	466.719	607.656	10.629	8.258	43,909.963	73,583.919	Distal ligament attachment	Distal ligament attachment
A-2	645.625	745.625	9.6295	7.202	67,046.576	103,530.27	Tibial avulsion	Distal ligament attachment
A-4	649.688	627.344	11.622	12.363	51,901.566	50,743.671	Femur metaphysis	Femur metaphysis
A-5	312.031	273.438	5.521	5.841	56,517.116	46,813.559	Tibial avulsion	Tibial avulsion

**Table 6:** Group B tensile test values

Subject	Ultimate tensile load (N)		Ultimate elongation (mm)		Stiffness (N/meter)		Failure mode	
	Experiment knee	Control knee	Experiment knee	Control knee	Experiment knee	Control knee	Experiment knee	Control knee
B-2	465.313	500.938	5.379	7.535	86,505.484	66,481.486	Tibial avulsion	Tibial avulsion
B-3	790.625	717.813	8.079	8.802	97,861.74	81,551.125	Tibial avulsion	Tibial avulsion
B-4	525	580.469	8.355	5.235	62,836,625	110,882.33	Distal ligament attachment	Distal ligament attachment
B-5	418.438	527.344	5.634	6.608	74,270.146	79,803.874	Tibial avulsion	Tibial avulsion

## Discussion

In our study, we aimed to investigate the primary healing capacity of the ACL tissue using the central defect type ACL injury model that was popularized by Murray et al. They performed and standardized this model for their studies for investigation of the primary healing and repair capacity of the ACL as compared to other ligaments and evaluation of healing enhancement methods with tissue engineering products [6, 10, 11]. They mainly concentrated on the histological healing process and emphasized that the model has healing capacity and that can be improved with tissue engineering products. In their studies, the biomechanical tests were only tensile loadings as they presumed the central defect model to be mechanically stabile, although they did not tested it. In the current study, the sagittal stability of the central defect model was confirmed by measuring ATT. Our macroscopic assessments of the samples did not show any cartilage injury that could have occurred in the setting of instability, further supporting the stability of the central defect model.

The previous studies using the central defect model did not specifically discuss the treatment of the fat pad tissue while creating the ligament defect. Such tissue may be important, as demonstrated in some ACL circulation studies [34–36]. In the perfusion study of Dunlap et al., ACL perfusion clearly decreased with division of the fat pad [35]. Our histological assessments on selected knee samples showed that after 10 weeks, the central defect was fully healed in fat pad protected sample. In the fat pad excised sample, we observed pathological findings such as myxoid degeneration, edema, and fibrosis. Fat pad excision disturbed the healing. We believe the blood supply to the ACL was decreased by excision of the fat pad, leading to a poorer healing response. As expected, no differences in anterior tibial translation were noted in this stable knee model, but we did note significant higher stiffness in the fat pad excision group in tensile loadings, which is thought to be caused by the fibrotic changes in this group that were observed during histological assessment.

The findings of this study have several potential applications. First, when utilizing this model in a research setting, care should be taken not to harm the infrapatellar fat pad as damage to this structure can alter the healing process and affect structural properties of the ligament, potentially confounding study results. Second, in a clinical setting, one should consider the role of the fat pad in ACL healing and consider preservation of as much tissue as possible during ACL reconstruction. Some recently published studies also emphasize the altered biologic responses in the fat pad after ACL reconstruction that can also be associated with poor outcome. In the study of Solbak et al., inflammational and fibrotic changes are found in the fat pad after ACL reconstruction on a sheep model [39]. Wang et al. also found fibrotic changes which are correlated with magnetic resonance imaging after ACL transaction on a rat model [40]. These studies also give information about the close biologic connection of the ACL and fat pad like our study. Although beyond the scope of our work, future studies could further explore the role of the fat pad also in the ligamentization process.

A limitation of this study is that we evaluated histology in only one sample from each group. The findings of this study can be further evaluated with studies that include more samples for histological analyses and with biomechanical correlation.

## Conclusion

The central defect type injury model was confirmed to be biomechanically stable in a goat model. Resection of the fat pad was noted to negatively affect defect healing and increase ligament stiffness in the central defect injury model.

### Ethical approval

The study was approved by the Ethic Commission of Ege University Animal Experiments Ethic Commission acceptance number 2010-134.

## Abbreviations

ACL: anterior cruciate ligament
ATT: anterior tibial translation
MCL: medial collateral ligament
mm: millimeters
UTL: ultimate tensile load
UE: ultimate Elongation
N: Newton
